# Determinants of woody encroachment and cover in African savannas

**DOI:** 10.1007/s00442-017-3807-6

**Published:** 2017-01-23

**Authors:** Aisling P. Devine, Robbie A. McDonald, Tristan Quaife, Ilya M. D. Maclean

**Affiliations:** 1 Department of Biosciences, Wallace Building, Swansea University, Singleton Park, Swansea, SA2 8PP UK; 2 Environment and Sustainability Institute, University of Exeter, Cornwall Campus, Penryn, TR10 9EZ UK; 3 Department of Meteorology, University of Reading, Earley Gate, PO Box 243, Reading, RG6 6BB UK

**Keywords:** Grassland, Shrub invasion, Rangeland, Carbon, Savannah

## Abstract

Savanna ecosystems are an integral part of the African landscape and sustain the livelihoods of millions of people. Woody encroachment in savannas is a widespread phenomenon but its causes are widely debated. We review the extensive literature on woody encroachment to help improve understanding of the possible causes and to highlight where and how future scientific efforts to fully understand these causes should be focused. Rainfall is the most important determinant of maximum woody cover across Africa, but fire and herbivory interact to reduce woody cover below the maximum at many locations. We postulate that woody encroachment is most likely driven by CO_2_ enrichment and propose a two-system conceptual framework, whereby mechanisms of woody encroachment differ depending on whether the savanna is a wet or dry system. In dry savannas, the increased water-use efficiency in plants relaxes precipitation-driven constraints and increases woody growth. In wet savannas, the increase of carbon allocation to tree roots results in faster recovery rates after disturbance and a greater likelihood of reaching sexual maturity. Our proposed framework can be tested using a mixture of experimental and earth observational techniques. At a local level, changes in precipitation, burning regimes or herbivory could be driving woody encroachment, but are unlikely to be the explanation of this continent-wide phenomenon.

**Electronic supplementary material:**

The online version of this article (doi:10.1007/s00442-017-3807-6) contains supplementary material, which is available to authorized users.

## Introduction

Over the past 60 years, growing evidence suggests that savannas throughout the world are being altered by a phenomenon known as ‘woody encroachment’ (Adamoli et al. 1990; Archer et al. 1995; Moleele et al. 2002). This changing balance in the proportion of trees and shrubs relative to grasses and herbs has been classed as a form of land-use degradation (Oldeland et al. 2010) and has been described as one of the dominant ecological changes in the last two centuries (Polley et al. 1997). One-fifth of the world’s population live within savanna regions and thus woody encroachment has important ecological and economic implications. The suppression of grasses and herbs by encroaching woody species, which are often unpalatable to domestic livestock, can have a negative impact on livelihoods (Kangalawe 2009). Changes in the composition of savannas are particularly important in Africa, which hosts a large and rapidly growing proportion of the world’s human population, many of whom are pastoralists (Scholes and Archer 1997). Woody encroachment has profound implications for biodiversity; it decreases landscape heterogeneity, reducing the diversity of invertebrates, birds and large mammals (Sirami et al. 2009; Smit and Prins 2015). Large-scale vegetation change also has consequences for energy, carbon and water budgets (Woodward and Lomas 2004; Mitchard and Flintrop 2013). Savanna ecosystems cover a high proportion of the global terrestrial land surface and thus have a significant role in earth–atmosphere feedback processes (Asner et al. 2003; Woodward and Lomas 2004; Bond 2008).

Drivers of woody encroachment in African savannas are still widely debated (Archer et al. 1995; Wigley et al. 2010). A research complexity within the woody encroachment literature is that most studies focus only on areas that are being encroached and ignore areas that are not. However a study by Mitchard and Flintrop (2013) examined both woody encroachment and woodland degradation in sub-Saharan Africa and demonstrated that woody encroachment was as prevalent as woodland degradation, thus showing that a bias in the literature towards woody encroachment is unlikely. Proposed drivers for woody encroachment fall broadly into two categories: local and global. A number of studies (e.g., Bond et al. 2003; Goheen et al. 2004; Higgins et al. 2007; Wigley et al. 2010) have helped to elucidate the causes of woody encroachment at specific locations and several studies have examined the determinants of woody cover from savanna sites across Africa (e.g. Sankaran et al. 2005, 2008). The wealth of recent information provides a timely opportunity to synthesise existing knowledge. In this review we critically examine the evidence in support of different hypotheses for woody encroachment. We aim to provide a coherent picture of woody encroachment, to propose a suite of hypotheses that could be used to guide future research, and propose how future scientific efforts should be focused to test these hypotheses.

## Global drivers

### Climatic interactions

Climate is a major force affecting the distribution of biomes. However, savannas often do not accord with bioclimate models, occurring in areas that could climatically support forests (Prentice et al. 1992; Bond et al. 2003). The likely reasons why savanna distribution conflicts with the results of bioclimate models are related to the localised effects of disturbance (Sankaran et al. 2005; Staver et al. 2011a, b; Lehmann et al. 2011; Murphy and Bowman 2012). Analyses of woody cover patterns across Africa suggest that maximum woody cover in savannas receiving a mean annual precipitation (MAP) of less than 650 mm is constrained by rainfall (Sankaran et al. 2005). These dry savannas are stable systems in which water constrains woody cover, permitting tree–grass coexistence. Although disturbances such as fire and herbivory do occur, they are not necessarily required to maintain a savanna system. In contrast, wet savannas receiving more than 650 mm mean annual precipitation (MAP) are unstable systems that require fire and herbivory in order to maintain a savanna system; these wet savannas would otherwise transform to a woodland system (Sankaran et al. 2005).

In more recent studies, this threshold of 650 mm MAP separating savannas into stable (dry) and unstable (wet) states has been questioned. Staver et al. (2011a, b) showed this threshold may be higher, at 1000 mm MAP. Staver et al. (2011b) examined tree cover in sub-Saharan Africa and in areas where rainfall was below 1000 mm MAP savannas were more likely to occur, whilst areas receiving over 2000 mm MAP were more likely to be dominated by forests. However, in areas receiving rainfall between 1000 and 2000 mm, both savannas and forests can occur, the presence of which depends on the prevalent fire or disturbance regime. Similarly Lehmann et al. (2011) showed that maximum woody cover is regulated by MAP and rainfall seasonality, but disturbance regulates open systems, maintaining these landscapes as either forest or savanna. However, Hirota et al. (2011) who conducted similar research to Staver et al. (2011b), using the same tree cover data but different precipitation data (Mayer and Khalyani 2011), found that the threshold of where tree cover/woody cover could exist was lower at 750 mm MAP, closer to threshold identified by Sankaran et al. (2005). Recent work by Viglizzo et al. (2015) supports that of Hirota et al. (2011), also finding a 750 mm MAP threshold. The confirmed lower threshold in this study in comparison to that identified by Staver et al. (2011b) was thought to be due to the separation of shrub land from closed canopy woodlands and the use of different precipitation data.

Murphy and Bowman (2012) proposed that savannas and forests could both exist in areas with the same climate as a result of interactions between tree growth and fire, with fire frequency determining the likelihood of an area being a savanna or a forest. Environmental factors such as increased rainfall or CO_2_ would increase tree growth and encourage a closed forest system. This in turn would lower grass fuel loads and increase humidity, which lowers the flammability of the landscape, creating a positive feedback system in maintaining a forest. In contrast, in areas where fire occurs more frequently, tree maturity rates decrease due to top-killing, which prevents canopy closure and allows C_4_ grasses to thrive (Mayer and Khalyani 2011). This in turn maintains aridity and increases grass fuel loads and thus flammability, ultimately maintaining an open savanna system. Hoffmann et al. (2012) also proposed similar ecosystem fire-growth responses for savannas in the Cerrado, whereby, two critical thresholds must be maintained for forests to occur rather than savannas. The first being the fire-resistance threshold, where trees have accumulated sufficient bark to avoid stem death. The second being the fire-suppression threshold, where there is sufficient canopy cover to suppress fire by excluding grasses. Ratnam et al. (2011) proposed similar mechanisms for regulating forest/savanna systems, though this study emphasised the importance of determining mesic savannas from degraded tropical forests. Overall, although rainfall availability is a key determinate of woody cover in savannas, knowledge of the interaction between climate and disturbance (predominantly fire) is essential to fully understand the distribution of savanna and forest systems.

It has been suggested that increased rainfall associated with climate change could be a driver for woody encroachment (Tews and Jeltsch 2004). Increased rainfall would affect dry savannas if mean annual rainfall were to exceed the 650–1000 mm threshold, as disturbance would then be required to maintain these systems (Sankaran et al. 2005; Staver et al. 2011a). However, unless rainfall were to exceed the critical threshold of 2000 mm MAP, increased rainfall in wet savannas would have minimal effect, as these systems are at a disequilibrium between vegetation and climate (Bond and Midgley 2012). Where rainfall exceeds 2000 mm MAP, closed forest systems tend to dominate regardless of disturbance (Staver et al. 2011b).

Changes in temperature could also affect interactions between rainfall and woody cover. Increases in temperature increase transpiration, effectively counteracting the effects of higher rainfall. Furthermore, Fensham et al. (2009) found that though increases in woody growth occurred in instances of increased rainfall, these were offset when extreme drought events occurred, causing widespread tree mortality. Climate change interactions in savanna systems can also vary at the species level. Though warming temperatures may have a negative impact on water use, it can also encourage germination in some tree species. Chidumayo (2007) demonstrated that seedling emergence and survival under elevated temperature varied among species. Seedling emergence increased for *Dichrostachys cinerea* but decreased for *Acacia polyacantha*, *Bauhinia thonningii* and *Ziziphus abyssinica*, whereas seedling mortality increased for *A*. *sieberana* and *D. cinerea* and decreased for *A. polyacantha, B. thonningii* and *Z. abyssinica.* Additionally, Stevens et al. (2014) demonstrated that higher temperatures reduce germination in *A. nigrescens* and *Colophospermum mopane* but result in slower seed bank depletion, while warmer conditions encourage radicle extension, increasing seedling establishment. However, not all savanna tree species have to reproduce by seed; many species, such as *D. cinerea,* reproduce vegetatively (Wakeling and Bond 2007). The effects on germination and changes in temperature are, therefore, likely to vary regionally, dependent upon specific traits and the existing species composition of the area.

Ascribing the major cause of woody encroachment to climate change is problematic not only because woody encroachment occurs in both wet and dry savannas, but also because changes in rainfall give rise to conflicting localised effects. February et al. (2013) demonstrates that localised increases in rainfall acted to increase competition between trees and grasses, ultimately suppressing tree growth. The establishment of trees occurred during periods of temporary droughts and intense grazing, as tree–grass competition was lowered in these circumstances. In contrast, Kulmatiski and Beard (2013) show that localised tree growth is not affected by rainfall quantity but by rainfall intensity, where trees outperformed grasses when rainfall intensities increased. Changes in climate will undoubtedly be important for woody growth and are a potential concern in dry savannas with limited disturbance. However, given the lack of spatial consistency in trends in precipitation across the globe, and the global extent of woody encroachment, it seems unlikely that change in rainfall patterns are the sole driver of woody encroachment.

### Soil type and nutrient availability

Savanna landscapes are known to overlay weathered, nutrient-poor soils associated with ancient land surfaces (Cole 1986). Thus, low nutrient availability is a widely cited hypothesis for the lack of trees in savanna biomes (Bond 2010), as poor soil quality limits tree growth. It has been suggested that woodland patches in savanna are associated with locally higher soil moisture and nutrient availability in areas known as “islands of fertility” (Mourik et al. 2007). However, the occurrences of trees in nutrient-rich soil may alternatively represent cause rather than effect, as the presence of trees improves soil quality, predominantly through nitrogen fixing processes and leaf litter accumulation (Hagos and Smit 2005). In contrast, other studies suggest that areas of high nutrient availability are associated with a reduction in woody cover, largely as a result of competition from grasses (Mills et al. 2013). Nutrient-rich soils associated with abandoned farmland often lack trees because seedlings are suppressed by the higher yields of grasses (van der Waal et al. 2011). Nonetheless, it has been shown that nutrient availability in soils can interact with rainfall to influence tree–grass dynamics. Lehmann et al. (2011) show that areas of low rainfall and high nutrient availability facilitate the growth of palatable grasses, which in turn leads to increased herbivory and the maintenance of a savanna system. In contrast, high rainfall and nutrient availability facilitate rapid tree growth, resulting in a transition to a forests system.

Soil nutrient availability and underlying geology may also alter vegetation communities through complex interactions with disturbance. In Kruger National Park woody cover was observed to increase in areas with nutrient-poor, granite soils and decrease in areas with nutrient-rich basalt soils over a sixty-year period, a pattern likely driven by herbivory (Eckhardt et al. 2000). It is thought that grasses in nutrient-poor soils do not recover as quickly under high grazing pressure as grasses in nutrient-rich soils, resulting in decreased tree–grass competition. The findings of Levick and Rogers (2011), from a more northerly section of Kruger are similar, but point to a more ubiquitous increase. Here, woody cover increased in both nutrient-poor granite soils and nutrient-rich basalt soils but the increase was slightly more prominent in the basalt areas due to presumed higher levels of grazing and lower fire frequency, which encouraged tree growth (Levick and Rogers 2011). In both of these studies all field sites were located in areas where MAP was below the wet savanna threshold, where tree–grass competition is much higher. It seems likely, therefore, that interaction between soil properties and disturbance has a more pronounced effect on dry savannas, as it reduces competition between trees and grasses (Sankaran et al. 2005). Buitenwerf et al. (2012) also demonstrate that woody encroachment occurs rapidly on granite-based soils but is predominantly absent in basalt-based soils. However, in this study, the location sampled on granite soils was also in unstable wet savanna, whereas the basalt-based study site was a dry stable savanna. Furthermore, Devine et al. (2015) observed much higher woody cover in a wet granite-based region than a dry granite-based area of Kruger National park. It would appear, therefore, that though interactions between soils properties and disturbance play an important role in regulating woody cover, the effects are more prominent in dry savannas.

### Carbon dioxide enrichment

Concentrations of atmospheric CO_2_ affect how plants function and grow, and in consequence increased atmospheric CO_2_ has been proposed as a driver for woody encroachment (Lloyd and Farquhar 1996; Ainsworth and Rogers 2007; Buitenwerf et al. 2012; Ward et al. 2014). Atmospheric CO_2_ concentrations have increased due to industrialisation from just above 280 ppm in the 1850s to over 400 ppm by 2013 (IPCC 2014). C_4_ plants use carbon more efficiently and evolved during periods of low atmospheric CO_2_. Photorespiration, which reduces the efficiency of photosynthesis, is more likely to occur in C_3_ plants during periods of high temperatures and low atmospheric CO_2_ (Sage 2004; Edwards et al. 2010). In contrast, C_4_ plants increase their carbon-fixation efficiency by saturating the Rubisco enzyme with CO_2_ (Ehleringer 1978; Edwards et al. 2010; Christin and Osborne 2014). Thus lower CO_2_ is competitively advantageous to C_4_ plants (Ehleringer et al. 1997; Edwards et al. 2010) and in eras of high atmospheric CO_2_, C_4_ plant have less of a competitive advantage over C_3_ plants offering a plausible reason for woody encroachment (Idso 1992).

The hypothesis of increased atmospheric CO_2_ concentrations as a global driver for woody encroachment would be broadly consistent with several studies both from glasshouse experiments and the field (Wigley et al. 2010; Hovenden and Williams 2010; Bond and Midgley 2012; Ward et al. 2014). Hovenden and Williams (2010), in their review of experimental evidence, identify 33 out of 36 different tree species growing on grasslands that showed an increase in growth with increased atmospheric CO_2_, compared to only 7 out 32 grass species that shows similar results. Long-term field studies also point to CO_2_ enrichment as a likely driver of woody encroachment, at least insofar as other likely drivers are eliminated: Wigley et al. (2010) demonstrate consistent woody encroachment in savannas with contrasting land tenure (commercial farming, conservation and communal rangeland). Their results show a significant increase in woody cover over 70 years across sites with similar climate, regardless of land use. Similar results can also be seen in Buitenwerf et al. (2012), where woody cover significantly increased over a 60-year period. Buitenwerf et al. (2012) examined woody cover at Kruger National Park where disturbance regimes had been kept constant over the same time period. Woody cover trebled over the 60 years (though some fluctuation was observed over time) despite the fact that disturbance regimes and rainfall had not changed.

However, the simplicity of the CO_2_ enrichment hypothesis has drawn criticism (Archer et al. 1995). Archer et al. (1995) suggest that increased atmospheric CO_2_ does not explain why C_3_ trees are replacing C_3_ grasses or why C_3_ grasslands are not replacing C_4_ grasslands. Edwards et al. (2010) hypothesised the expansion of C_4_ grasses over C_3_ grasses were not solely due to different photosynthetic pathways. In this phylogenetic study of C_3_ and C_4_ grasses it was proposed that a group of C_4_ grass species had traits promoting species dominance, such as protected buds, storage reserves, slow decomposition and quick resprouting rates after herbivory, all of which could have facilitated C_4_ dominance (Edwards et al. 2010). Thus, the theory that C_4_ plants no longer have a competitive advantage over C_3_ plants due to increasing atmospheric CO_2_ appears to be an over simplification. In addition, work by Miller-Rushing et al. (2009) showed that C_3_ plants species exhibit highly variable physiological responses to increasing atmospheric CO_2_. Furthermore Archer et al. (1995) also highlight that CO_2_ enrichment does not explain why some savannas are not experiencing woody encroachment, or are experiencing tree decline or desertification instead (Fensham et al. 2009; Lehmann et al. 2011). The criticisms of Archer et al. (1995) are founded mainly on the assumption that CO_2_ enrichment simply lowers competition between trees and grasses, favouring woody growth. However, increased atmospheric CO_2_ can also alter plant physiological responses other than those resulting from different photosynthetic pathways (Bond and Midgley 2000; Hetherington and Woodward 2003), which can indirectly promote woody encroachment.

Increased atmospheric CO_2_ can decrease stomatal conductance in plants, which reduces transpiration, increasing water-use efficiency (Polley 1997). Decreases in stomatal conductance through increased atmospheric levels of CO_2_ can occur across plant groups in both trees and grasses regardless of photosynthetic pathways (Lloyd and Farquhar 1996; Drake et al. 1997; Norby and Zak 2011), though C_3_ and C_4_ grasses are usually shown to be the most responsive to this (Ainsworth and Long 2005). Ultimately a decrease in stomatal conductance leads to higher moisture availability in soils. One would therefore expect rising CO_2_ concentrations to slow the depletion of soil moisture by grasses, thus favouring shrubs and trees that would otherwise succumb to water stress (Polley et al. 1997). However, evidence for interacting effects between water availability and CO_2_ on woody encroachment is less conclusive.

Localised increases in water availability in savannas have shown contrasting and variable effects on tree growth (Riginos 2009; Ward and Esler 2010; Kulmatiski and Beard 2013; February et al. 2013; Kambatuku et al. 2013). Increased water availability in soils may not result directly in increased woody growth as it can increase competition from grasses, as shown by February et al. (2013). Work by Ward and Esler (2010) showed that encroachment by *Acacia* species is suppressed by water competition from grasses. Furthermore, recent work by Manea and Leishman (2015) showed that, under elevated CO_2_ conditions, the growth of C_4_ grasses increases greatly, resulting in greater leaf area. This in turn, restricts light and water to tree seedlings, suppressing their growth and offsetting the benefits provided by increased moisture availability resulting from reduced stomatal conductance. Moreover, though increased atmospheric CO_2_ is known to reduce stomatal conductance, research by Zeppel et al. (2011) shows that nocturnal stomatal conductance increases under elevated CO_2_. This can leave plants vulnerable to drought and CO_2_ induced stomatal changes could thus have an adverse impact on woody growth.

Assuming that increased CO_2_ does have a positive impact on water-use efficiency, this effect could be cancelled out by changes in average temperatures. Additionally, leaf area index is known to increase under elevated CO_2_, offsetting additional water availability that results from reduced stomatal conductance (Cheng et al. 2014). Angert et al. (2005) demonstrate that in the northern hemisphere, any benefit plants experience from increased CO_2_ is counteracted by increasing summer temperatures. Furthermore, while plant growth in free air carbon dioxide enrichment (FACE) experiments shows an increase under elevated CO_2_, it is lower than expected due to the effects of rising temperatures (Ainsworth and Long 2005). In relation to savanna regions that experience temperature increases, transpiration and water stress will also increase. It is possible, therefore, that any additionally water saved through decreases in stomatal conductance would be used to mitigate against this added stress, with the net consequence that there is little effect on tree and grass growth rates. In consequence, predicting the overall effects of increased soil moisture from a decrease in stomatal conductance is made challenging by different stomatal responses and confounding interacting factors, such as plant traits, composition and local climate (Cheng et al. 2014).

The dynamics of tree–grass interactions make it difficult to determine the exact response of tree growth to localised increases in water availability. Additionally, increases in water availability caused by CO_2_ enrichment would not fully explain woody encroachment in savannas where water availability is not a limiting factor to woody growth (Sankaran et al. 2005; Staver et al. 2011a, b; Lehmann et al. 2011; Murphy and Bowman 2012). In savannas that are constrained by water availability, the indirect effect of increased water availability through decreased stomatal conductance may relax these constraints, increasing woody growth. However, localised variation in tree–grass competition makes it difficult to fully determine the effects of reduced stomata conductance on woody encroachment.

Increased atmospheric CO_2_ can also cause an increase in the allocation of carbon to below-ground biomass, providing additional energy to trees to re-sprout after a disturbance (Bond and Midgley 2000). This proposed mechanism could be crucial in tipping any demographic balance between tree–grass coexistence in relation to burning regimes. Trees are often suppressed at a sapling stage for decades due to burning and browsing (Higgins et al. 2000; Sankaran et al. 2013); these trees are not juveniles but growth-suppressed individuals known as “Gullivers” (Bond and Wilgen 1996; Higgins et al. 2000; Bond 2008). Therefore, if trees have higher rates of resprouting after fire, individuals are more likely to escape the fire zone and decrease their chance of being top-killed, which will lead to increases in tree recruitment. This hypothesis is supported by evidence from savanna species such as, *A. karroo* and *A. nilotica,* which show increased allocation of carbon to rootstocks under elevated CO_2_, resulting in faster growth after disturbances (Wigley et al. 2009; Kgope et al. 2010). Many non-savanna plant species have exhibited similar responses. Solanaceae plants, for example, show an increase in tubers per individual (Miglietta et al. 1998). Increased allocation of carbon rootstocks is often observed in areas of lower resource competition. Hoffmann et al. (2000) saw diminished effects of carbon root allocation in the cerrado savanna when nutrient availability was limited. Furthermore, a decrease in rootstock carbon was observed in *A. karroo* when constant defoliation was applied (Schutz et al. 2010). Therefore, increased allocation of carbon to rootstocks through increased atmospheric CO_2_ appears to have the largest impact in regions where trees have lower competition for essential resources, such as water and nutrients, as additional energy is available to replenish root carbohydrate stocks (Schutz et al. 2009). Thus, increased allocation of carbon to rootstocks may have a more significant impact in wet savannas than in dry savannas.

Elevated CO_2_ can also stimulate overall plant growth, which has important implications for the rate at which savanna trees escape the fire surface zone. Increased growth under elevated CO_2_ has been shown for non-savanna trees, such as *Quercus ilex* (Hattenschwiler et al. 1997), though this accelerated growth was most prominent where intra-species competition for light was decreased. Additionally, Liberloo et al. (2005) observed negligible increases in above ground biomass in poplar trees under conditions of elevated CO_2_. It would appear, therefore, that although increases in atmospheric CO_2_ increase overall tree growth, it is increases in carbon rootstocks that have greater influence on savanna tree recovery after fire.

Another possibility is that trees become more predisposed towards reproducing vegetatively through clonal root systems through increased allocation of carbon to rootstocks. Vesk and Westoby (2004) and Bond and Midgley (2012) also highlight the importance of fire in selecting tree species with clonal root spreading systems that recover rapidly after fire as a mechanism driving encroachment. Trees with root systems that have large carbons stocks are known to be more responsive to increased carbon dioxide, and respond more quickly and store surplus CO_2_ more easily (Wakeling and Bond 2007; Bond and Midgley 2012). Thus, if increased allocation of carbon to rootstocks increases the ability of root cloning, this may be a plausible explanation of the expansion of wooded patches on savannas.

**Fig. 1:**
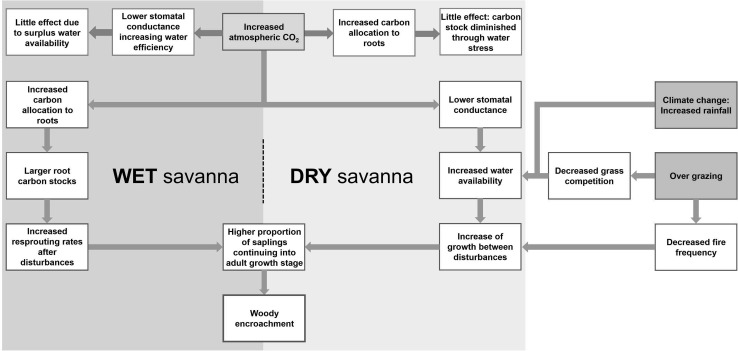
Overview of mechanisms hypothesised to influence woody encroachment. *Grey shaded boxes* represent extrinsic drivers and the *white shaded boxes* illustrate the effects of these drivers. *Boxes outlined in red* indicate intrinsic factors that constrain woody encroachment and their effect, the *boxes and arrows outlined in green* illustrate biotic processes and physiological mechanisms leading to woody encroachment. Woody encroachment in wet savannas is proposed to result from increased carbon allocation to rootstocks, which rapidly increases recovery after disturbances allowing higher proportion of trees to reach reproductive maturity. This in turn facilitates a positive feedback in favour of trees by increasing tree recruitment. Conversely, in dry savannas, increased carbon allocation to rootstocks is diminished by water stress. In dry savannas, which are constrained by water, increased water efficiency, driven by increased CO_2_, promotes additional tree growth thus allowing more trees to reach sexual maturity. However, the rate and magnitude of woody encroachment in dry savannas will be affected by localised factors of rainfall and grazing, which both alter water availability. It is, therefore, likely that woody encroachment in dry savannas will be less rapid than in wet savannas, though the magnitude of woody encroachment in wet savannas will be affected by localised fire regimes (color figure online)

Overall, the interactions between CO_2_ and woody growth are complex and not easily consolidated into one overarching hypothesis. It would appear that elevated CO_2_ alters woody growth and function depending on whether or not a savanna is situated in a dry or wet region (Fig. 1). In semi-arid regions it would appear that decreased stomatal conductance may alleviate water competition increasing woody growth, but the localised effects may vary depending on disturbance, grass competition and localised climate change. Woody encroachment in wetter regions appears to be facilitated through an increase of carbon allocation to root biomass, which in turn increases resprouting rates after disturbance. It is likely, however, that the precise mechanisms vary depending on species-specific resprouting traits and resource competition for nutrients, light and water.

## Local drivers

### Fire regimes

Changes in fire management practices have also been proposed as a driver of woody encroachment (O’Connor et al. 2014). Savanna biomes experience frequent fires, and some savannas are classed as a fire-dependent system (Bond et al. 2005). Fire pre-dates human interference (Cahoon et al. 1994), although most fires are currently caused by people (Roy et al. 2002; Eriksen 2007). Fires on savannas are used as a management tool to suppress woody cover and to achieve specific ecological outcomes (Parr and Andersen 2006). Fire lowers woody biomass in savannas by suppressing tree recruitment and lowering tree abundance (Higgins et al. 2000; Grady and Hoffmann 2012). Under frequent or intense burning regimes, seedling and sapling establishment is limited and trees are often suppressed from reaching a reproductive stage (Bond and Wilgen 1996; Bond and Midgley 2000). Once lowered, the lack of trees makes a savanna more susceptible to subsequent burning, helping to maintain them as open systems (Lehmann et al. 2011). However, fire rarely causes total tree mortality, with the exceptions of very intense fires (Hoffmann and Solbrig 2003). Mature adult trees that have grown out of the fire zone usually receive only superficial fire damage, such as charred bark and burnt leaves (Higgins et al. 2000). Sapling trees that occur within the fire zone are top-killed instead and can then resprout again from the roots (Higgins et al. 2000; Ryan and Williams 2011). These trees, otherwise known as “Gulliver trees”, can spend decades trapped within the fire zone not being able to resprout quickly enough before the next fire (Higgins et al. 2000). On a landscape level, fire decreases woody cover below its potential maximum (Sankaran et al. 2005), but on a localised level tree species will respond differently to fire depending on trait differences (Higgins et al. 2012), which may be responsible for the localised heterogeneity often observed in savannas (Scholtz et al. 2014).

Changes in fire regimes are spatially varied and complete fire exclusion in African savannas is rare (Cahoon et al. 1994). Changes in fire frequency and seasonality will have varied effects on woody cover, as changes in frequency and timing cause variation in intensity (Smit et al. 2010). Frequent fires are usually less intense than infrequent fires due to smaller accumulation of grass fuel loads. Late dry season fires can be more damaging then wet season fires due to drier and larger grass fuel loads (Govender et al. 2006). Thus, the extent to which tree–grass interactions and feedbacks are altered by fire greatly depends on the seasonality, frequency and magnitude of burning regimes (Govender et al. 2006; Merino-de-Miguel et al. 2010; Smit et al. 2010). The issue is further complicated by tree species displaying varying degrees of fire tolerance, which can shift species composition and abundance (Higgins et al. 2012). Species such as, *A. polyacantha* and *A. sieberiana*, for example require fire-induced seed scarification for successful germination (Chidumayo 2008). Observations have shown *A. sieberiana* to colonise quickly after a burn, increasing woody biomass in savanna landscapes. Additionally, fire can promote woody pioneer species in some circumstances (Bucini and Hanan 2007) and species such as, *D. cinerea* can reproduce rapidly after fire through vegetative reproduction (Wakeling and Bond 2007). Vegetation responses to changes in fire regimes will thus depend on the fire tolerance of the individual species that make up savanna communities.

The management of fire has changed greatly over the last century. During the period of European colonisation of Africa, fire was perceived as damaging and so the frequency of fire decreased and its seasonality changed (Eriksen 2007). Specifically in South Africa, fire management strategies in national parks have changed greatly over the last century (Bond and Archibald 2003; van Wilgen et al. 2014). Fire regimes changed from being strictly regimented to lightning strike policies (Du Toit et al. 2003) all of which led to an unwanted ecological state or to uncontrollable wild fires (Mentis and Bailey 1990). At present, most fire regimes are implemented to promote pyrodiversity and spatial heterogeneity (Parr and Andersen 2006). It is undeniable that fire regimes have varied over time, which may have historically encouraged woody encroachment, especially if fire regimes were excluded in areas for long periods of time. This is demonstrated by the results of fire exclusion experiments, which have resulted in a complete biome switch in wet savannas to closed forest systems (Trapnell 1959). However, fire–vegetation dynamics can display great heterogeneity in space and time and so quantifying and analysing spatial changes in fire regimes is difficult due to the lack of long-term records. Although it is evident that tree–grass dynamics are significantly affected by fire, it is difficult to propose a coherent theory of the influence of fire and woody encroachment on a global scale.

### Herbivory

Enhanced grazing has also been proposed as a driver of woody encroachment (Archer et al. 1995), especially in dry savannas (Tefera et al. 2007; Graz 2008; Auken 2009). Increased grazing reduces grass cover, which decreases the level of competition for water experienced by tree seedlings (Belsky and Blumenthal 1997; Goheen et al. 2004). Field experiments conducted by February et al. (2013) have identified the importance of grazing in lowering competition and promoting woody growth. Furthermore, increased grazing in dry savannas alters grass composition from perennial to annual species, which can alter soil moisture availability, again favouring the establishment of woody species (Graz 2008). Grazing not only decreases the competitive environment for tree seedlings, it also lowers the frequency of natural fires and lowers their intensity due to lower fuel loads, all of which promotes woody encroachment (Auken 2009). It would appear that in dry savannas, where water availability is the over-riding factor limiting woody growth, intensified grazing increases water availability and promotes the establishment of woody species.

In common with changing fire management, grazing patterns have changed over the last century. Herbivory, specifically grazing, occurs in all savannas, by both wild animals and livestock. Sub-Saharan Africa has experienced rapid human population increase over the last century, which has greatly increased the demand for food derived from livestock (Darkoh 2009). This could explain, at least on a regional level, why woody encroachment has occurred in areas of increased grazing. Paradoxically, however, continental-scale analyses of the processes that control woody encroachment suggest that higher cattle densities result in lower woody cover (Bucini and Hanan 2007), though this pattern co-occured in the presence of changing fire regimes and human population densities.

An added complexity associated with woody encroachment in African savanna is the role of wild mega herbivores, specifically elephants *Loxodonta africana*, which consume large volumes of biomass and are characterised as ecosystem engineers (Caughley 1976). Browsers rather than grazers have been known to suppress woody establishment creating a “browser-trap” that is functionally similar to the “fire trap” (Sankaran et al. 2013). Thus, the removal or local extinction of browsers has been proposed to cause woody encroachment (Sankaran et al. 2013). Natural browser populations, especially elephants, have decreased considerably in Africa over the last century due to human exploitation and habitat fragmentation (Du Toit et al. 2003). Changes in natural populations of both grazers and browsers coincide with woody encroachment, although this pattern may not be applicable at a global scale, due to the stochastic nature of wild animal populations and the absence of mega-fauna outside of Africa. Due to increased conservation efforts in sub-Saharan Africa over the last 20 years, wild mega-fauna, mainly elephants, have increased dramatically, particularly in protected areas (Asner et al. 2009; Lovett 2009), yet woody encroachment is still occurring at these locations (Buitenwerf et al. 2012). The degree of herbivory is a highly important savanna management issue and appears to be most influential in dry savanna regions. At a local level, the effects of herbivory may interact with other drivers and should be considered in any coherent hypothesis for woody encroachment.

## Conclusion: towards a coherent hypothesis for woody encroachment

The reasons for woody encroachment are still something of a puzzle. It is likely that multiple drivers interact to cause woody encroachment. The uncertainty lies mainly in quantifying the importance of these drivers and understanding the extent to which they interact with one another. Factors such as herbivory, fire, and soil properties are likely to alter woody cover and rates of encroachment in both wet and dry savannas at a local level. Overall, however, we propose the most plausible driver of woody encroachment on a global level is increased atmospheric CO_2_, as the other putative causes are not sufficiently ubiquitous in time and space to explain the degree of woody encroachment. There are crucial differences between dry (stable) and wet (unstable) savannas, particularly with regards to the role that water plays in altering the tree–grass competition (Sankaran et al. 2005). Thus, we propose that CO_2_ driven woody encroachment in both wet (unstable) and dry (stable) savannas are caused by two separate mechanisms (Fig. 1).

We thus propose a two-system conceptual framework of how woody encroachment is occurring (Fig. 1), whereby mechanisms of woody encroachment differ depending on whether the savanna is a wet or dry system. In dry savannas, where water availability is the most limiting factor for tree growth, we propose that CO_2_ enrichment is causing woody encroachment by increasing plant water-use efficiency, reducing the depletion of soil moisture, thus lowering precipitation-driven constraints in maximum woody cover. Enhanced water efficiencies causes increased water availability, encouraging higher growth rates, which in turn allows larger proportions of tree population to reach adult maturity, thus increasing rates of tree recruitment. However, it is important to note that localised changes in grazing and climate influence water availability will thus affect the degree of woody encroachment.

In wet savannas, CO_2_ induced water-use efficiency would have limited impact due to existing surplus water availability, and we propose instead that woody encroachment is caused by CO_2_ enrichment increasing the allocation of carbon to rootstocks in trees. Greater allocation of carbon to rootstocks increases the rates of height accumulation after and between fire and disturbances. This in turn creates a higher proportion of adult trees, again increasing tree recruitment. Furthermore, we propose that this mechanism has greatest effect in areas of decreased resource competition for water and nutrients and where trees experience less stressful incidents such as drought and defoliation. In these savannas, where water competition is lower, woody vegetation is in a better position to take advantage of allocating additional carbon to roots. Therefore wet savannas are most vulnerable to the effects of CO_2_ enrichment causing woody encroachment, which is also in line with work by Bond and Midgley (2012). In both systems, however, it is the existence of a positive feedback whereby the presence of fire increases their susceptibility to subsequent fires that makes them especially sensitive to CO_2_ enrichment, as this in turn influences the ease with which trees can escape the ‘fire trap’.

In this review we provide a new conceptual framework for woody encroachment  that may be tested using the following approaches. Firstly, the application of earth observational techniques using remote sensing data, such as vegetation index products combined with global climate data (see Huete et al. 2002 and Harris et al. 2014 for reviews respectively), can be used to observe the extent of woody encroachment across wet and dry savannas and examine whether or not rates of encroachment differ between these two different savanna systems. Furthermore burned area products (see e.g. Justice et al. 2002 for a review) can be applied to examine if recovery rates after fire have increased over time at a regional scale. The application of earth observation approaches would directly allow a comparison of woody encroachment in dry and wet savanna systems; however, it would not allow a direct examination of plant responses to increased atmospheric CO_2_.

The alternative to using earth observations would be to apply an experimental approach. The artificial manipulation of CO_2_ concentrations using FACE approaches (see, e.g., Ainsworth and Long 2005 for a review) would, if conducted at a dry location with experimental addition of water to a subset of plots, allow the direct physiological responses of savanna trees to be measured. It would, therefore, be possible, using porometer measurements for example, to directly measure stomata conductance under varying levels of CO_2_ (see, e.g., McDermitt 1990 for a review of approaches). It would also be possible to test directly whether carbon allocation to root stocks increases under-elevated CO_2_, by simply measuring below-ground biomass accumulation under wet savanna conditions, similar glasshouse experiments have been conducted in the past (Kgope et al. 2010). A significant benefit of an experimental approach would be that it is much more feasible to demonstrate a direct causal link between elevated CO_2_ and plant physiological responses, but major disadvantages include the potential length of time it takes for these physiological responses to translate into woody encroachment. Particularly in Africa, where securing funding for research may be challenging, the implementation of long-term FACE experiments is likely to be problematic. It is no coincidence that elevated CO_2_ experiments are highly concentrated around North American and European ecosystems, despite the fact that it is tropical terrestrial ecosystems that contribute most to global carbon budgets (Jones et al. 2014). To fully understand the causes of woody encroachment in African savannas, a readdress of this balance is needed.

Additionally the natural increases in CO_2_ concentrations in long-term field experiments could also be exploited, in effect conducting a natural experiment. However, rather than simply measuring the degree of woody encroachment at a variety of locations, as is often done at present, researchers should be encouraged to implement approaches that allow potential causal mechanisms to be invested with greater rigour. A key to achieving this would be measure plant physiological responses. Again, however, it is the time-scales over which one can expect physiological response to translate into ecosystems responses that make such an approach challenging. Although not without precedent in Africa, as demonstrated, for example, by the long-term fire-exclusion experiments at Kruger National Park (e.g., Devine et al. 2015), it is rare for plant physiological responses to be included among the suite of field measurements taken as part of these experiments. Nonetheless, resolving the puzzle of woody encroachment (almost by definition), requires that savanna ecosystem responses to changing conditions can be understood and predicted. Additionally examining dry savanna regions that are not experiencing woody encroachment could shed light on the most important drivers causing woody encroachment in dry savanna systems. While it is always possible to propose sophisticated hypotheses and predictive models, it is only by testing these against data, that these can be convincingly validated. Until long-term experiments that investigate the causes have been carried out, it is unlikely that the phenomenon of woody encroachment will be fully understood.

Overall, however, we propose that increased atmospheric CO_2_ is the over-riding factor driving woody encroachment, though the ecological mechanisms involved differ depending on whether the savanna in a wet or a dry system. At a local level, changes in precipitation, burning regimes or herbivory could be driving woody encroachment, but are unlikely to be the explanation of this global phenomenon.

## Electronic supplementary material

Below is the link to the electronic supplementary material.

Supplementary material 1 (PDF 255 kb)
