# Supplementary material for: Determinants of woody encroachment and cover in African savannas
Source: Oecologia. 2017 Jan 23;183(4):939–51. doi: 10.1007/s00442-017-3807-6 (PMC5348564; doi:10.1007/s00442-017-3807-6)
Supplement: Supplementary file 1 — Supplementary material 1 (PDF 255 kb) [file 442_2017_3807_MOESM1_ESM.pdf]

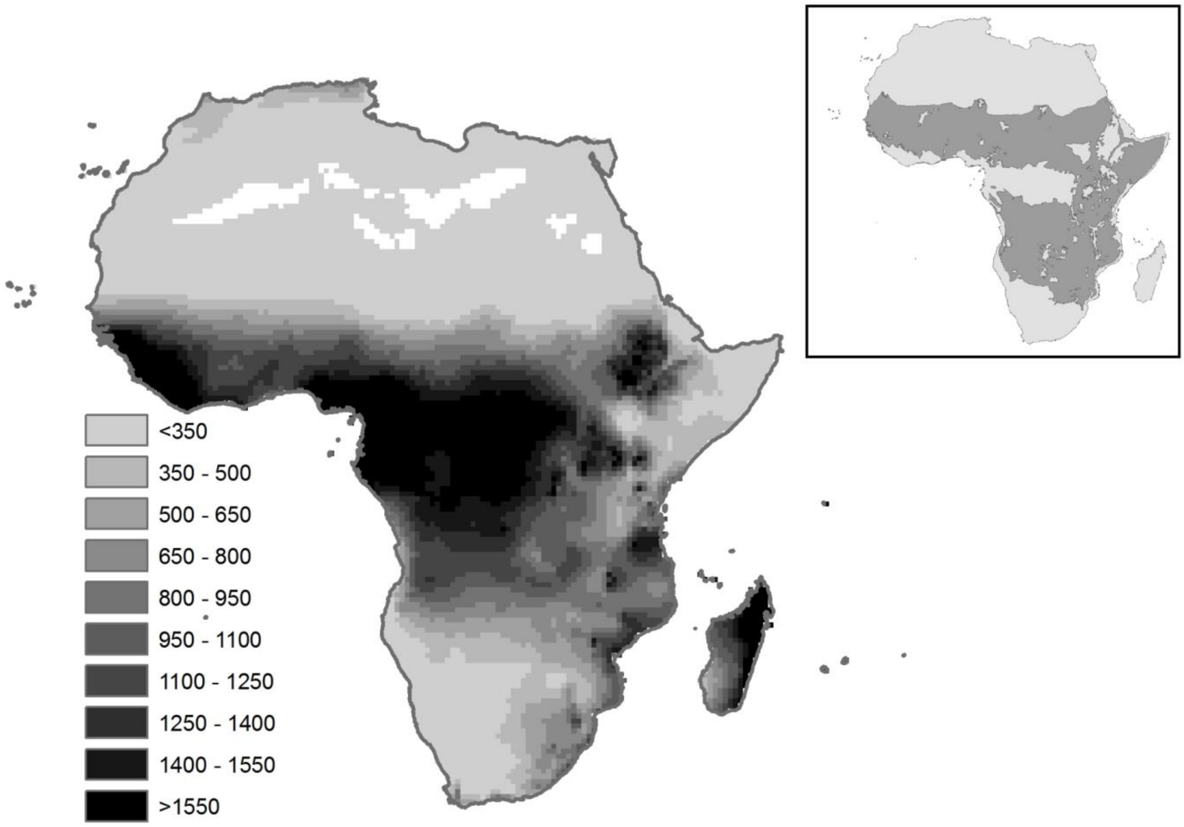

Mean annual rainfall (mm) for Africa, data derived from (Harris et al. 2014). Inset map displays the extent of savanna distribution in Africa, data derived from (Olson et al. 2013).

References

Harris I, Jones PD, Osborn TJ, Lister DH (2014) Updated high-resolution grids of monthly climatic observations - the CRU TS3.10 Dataset. Int J Climatol 34:623–642. doi: 10.1002/joc.3711

Olson DM, Dinerstein ED, Wikramanayake ND, et al (2013) Terrestrial Ecoregions of the World: A New Map of Life on Earth. Bioscience 51:933–938.
